# Correction: Hameed et al. Green Synthesis of Zinc Oxide (ZnO) Nanoparticles from Green Algae and Their Assessment in Various Biological Applications. *Micromachines* 2023, *14*, 928

**DOI:** 10.3390/mi16090995

**Published:** 2025-08-29

**Authors:** Hajra Hameed, Abdul Waheed, Muhammad Shakeeb Sharif, Muhammad Saleem, Afshan Afreen, Muhammad Tariq, Asif Kamal, Wedad A. Al-onazi, Dunia A. Al Farraj, Shabir Ahmad, Rania M. Mahmoud

**Affiliations:** 1 Department of Biotechnology, Mirpur University of Science and Technology, New Mirpur City 10250, Pakistan; hajrahameedmughal@gmail.com (H.H.);; 2 Agricultural Genomics Institute at Shenzhen, Chinese Academy of Agricultural Sciences, Shenzhen 518120, China; 2021y90100044@caas.cn; 3 Department of Plant Sciences, Quaid-i-Azam University, Islamabad 45320, Pakistan; 4 Department of Chemistry, College of Science, King Saud University, Riyadh 11495, Saudi Arabia; 5 Department of Botany and Microbiology, College of Science, King Saud University, Riyadh 11495, Saudi Arabia; 6 Department of Botany and Biodiversity Research, University of Vienna, 1010 Vienna, Austria; 7 Department of Botany, Faculty of Science, University of Fayoum, Fayoum 63514, Egypt

Following publication, concerns were raised regarding the peer-review process related to the publication of this article [1]. Adhering to our standard procedure, the Editorial Board conducted an investigation which determined that, while the peer-review process did comply with MDPI’s Editorial process policy (https://www.mdpi.com/editorial_process), the contribution of one of the three reviewers did not comply with MDPI’s Guidelines for Reviewers (https://www.mdpi.com/reviewers#_bookmark11) and the expectations of the Editorial Board. 

As a result, the Editorial Board has decided to remove the contribution of one of the reviewers from the open peer-review record (https://www.mdpi.com/2072-666X/14/5/928/review_report). In line with this, one reference originally included at the reviewer’s suggestion has also been removed from the article.

The authors have made the following revisions to this article [1]:

**References**

As originally recommended by the reviewer, reference 13 has been removed:

13. Nosrati, H.; Khodaei, M.; Banitalebi-Dehkordi, M.; Alizadeh, M.; Asadpour, S.; Sharifi, E.; Ai, J.; Soleimannejad, M. Preparation and characterization of poly (ethylene oxide)/zinc oxide nanofibrous scaffold for chronic wound healing applications. *Polym. Med.* **2020**, *50*, 41–51.

The authors state that the scientific conclusions are unaffected. This correction was approved by the Academic Editor. The original publication has also been updated.
